# Prognostic relevance of high expression of kynurenine pathway markers in glioblastoma

**DOI:** 10.1038/s41598-024-65907-3

**Published:** 2024-06-28

**Authors:** Arnaud Jacquerie, Ann Hoeben, Daniëlle B. P. Eekers, Alida A. Postma, Maxime Vanmechelen, Frederik de Smet, Linda Ackermans, Monique Anten, Kim Severens, Axel zur Hausen, Martinus P. G. Broen, Jan Beckervordersandforth

**Affiliations:** 1 Department of Pathology, GROW School for Oncology and Reproduction, Maastricht University Medical Centre, Maastricht, The Netherlands; 2 Department of Medical Oncology, GROW School for Oncology and Reproduction, Maastricht University Medical Centre, Maastricht, The Netherlands; 3 Department of Radiation Oncology (Maastro), GROW School for Oncology and Reproduction, Maastricht University Medical Centre, Maastricht, The Netherlands; 4 Department of Radiology and Nuclear Medicine, School for Mental Health and Neuroscience, Maastricht University Medical Centre, Maastricht, The Netherlands; 5 Laboratory for Precision Cancer Medicine, Translational Cell and Tissue Research Unit, Department of Imaging and Pathology, KU Leuven, Leuven, Belgium; 6 LISCO—KU Leuven Institute for Single Cell Omics, KU Leuven, Leuven, Belgium; 7 Department of Neurosurgery, School for Mental Health and Neuroscience, Maastricht University Medical Centre, Maastricht, The Netherlands; 8 Department of Neurology, GROW School for Oncology and Reproduction, Maastricht University Medical Centre, Maastricht, The Netherlands

**Keywords:** Kynurenine, IDO, TDO2, AhR, Prognosis, Glioblastoma, Prognostic markers, CNS cancer

## Abstract

Glioblastoma (GBM) continues to exhibit a discouraging survival rate despite extensive research into new treatments. One factor contributing to its poor prognosis is the tumor's immunosuppressive microenvironment, in which the kynurenine pathway (KP) plays a significant role. This study aimed to explore how KP impacts the survival of newly diagnosed GBM patients. We examined tissue samples from 108 GBM patients to assess the expression levels of key KP markers—tryptophan 2,3-dioxygenase (TDO2), indoleamine 2,3-dioxygenase (IDO1/2), and the aryl hydrocarbon receptor (AhR). Using immunohistochemistry and QuPath software, three tumor cores were analyzed per patient to evaluate KP marker expression. Kaplan–Meier survival analysis and stepwise multivariate Cox regression were used to determine the effect of these markers on patient survival. Results showed that patients with high expression of TDO2, IDO1/2, and AhR had significantly shorter survival times. This finding held true even when controlling for other known prognostic variables, with a hazard ratio of 3.393 for IDO1, 2.775 for IDO2, 1.891 for TDO2, and 1.902 for AhR. We suggest that KP markers could serve as useful tools for patient stratification, potentially guiding future immunomodulating trials and personalized treatment approaches for GBM patients.

## Introduction

Glioblastoma (GBM) is characterized by its highly malignant nature, intrinsic therapy resistance, and inevitable recurrence^1^. Even with comprehensive multimodal treatments involving surgery, radiotherapy, and chemotherapy, the median survival remains disappointingly limited to 14–16 months^1^. Their treatment resistance is, among others, attributed to the immunosuppressive tumour microenvironment in GBM^2^, which is also postulated as a possible explanation for the recent failure of immunotherapy studies^3,4^.

**Figure 1:**
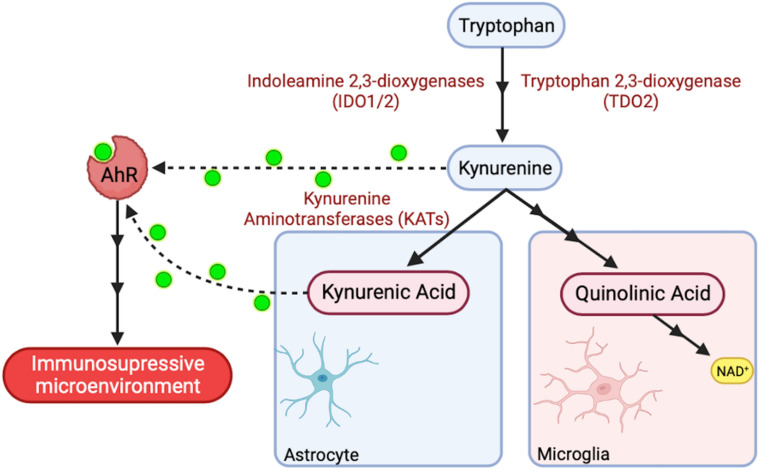
Simplified illustration of the kynurenine pathway (KP). Kynurenine and kynurenic acid are endogenous ligands of the aryl hydrocarbon receptor (AhR). Solid lines with multiple arrowheads symbolize the step-by-step involvement of additional enzymes. Dotted lines indicate the activation of AhR by kynurenine and kynurenic acid. The activation of AhR by kynurenine ligands is believed to mediate immunosuppression. Created with BioRender.

Recently, the kynurenine pathway (KP) has entered the stage as a key regulator of the tumour immune microenvironment in GBM^5,6^. The KP represents a metabolic pathway (Fig. 1) responsible for the degradation of the essential amino acid tryptophan (TRP), giving rise to a variety of bioactive compounds known as kynurenines (KYN)^7,8^. Under normal conditions, TRP is metabolized into KYN by the isoenzymes tryptophan 2,3-dioxygenase (TDO2) and indoleamine 2,3-dioxygenase (IDO) 1 and 2^8,9^. However, in GBM, the KP undergoes profound dysregulation. This dysregulation leads to substantial local depletion of tryptophan and an overexpression of IDO1/2 enzymes in GBM tissue, consequently resulting in the accumulation of kynurenine metabolites^8,10^. Kynurenines serve as endogenous ligands for the aryl hydrocarbon receptor (AhR), a cytoplasmic transcription receptor linked to carcinogenesis^8,11^. AhR plays a crucial role as a master regulator, controlling the differentiation and effector functions of T cells, lymphocytes, and macrophages^10–15^. Therefore, activation of AhR by kynurenine ligands is believed to be a major contributor to the immunosuppressive environment observed in GBM^10–15^.

As a consequence, expression level of genes of the KP is hypothesized as a surrogate of tumor altered immune microenvironment status and therefore postulated to be associated with a more aggressive disease course and worse survival^8,16^.

Our study aimed to examine the expression of the key KP proteins TDO2, IDO1/2 and AhR, in newly diagnosed glioblastoma patients and their correlation with survival.

## Methods

### Patients

From an existing genotyped glioma database covering routine clinical diagnostics, data and tissue samples from 135 glioblastoma patients diagnosed or treated in Maastricht University Medical Center + (MUMC+, the Netherlands) between 2006 and 2014 were available^17^. For our study, only patients with newly diagnosed glioblastoma (WHO grade 4), isocitrate dehydrogenase gene 1 or 2 (IDH1/2) wildtype and MGMT hypermethylated or unmethylated were included. Patient characteristics and clinical data were retrieved from medical records. Alongside demographic characteristics, also known prognostic factors^18^: ECOG Performance Status at baseline (score 0–4), corticosteroid use at baseline (yes/no), type of surgery (biopsy only or resection) and first-line glioblastoma treatment (type of treatment, early discontinuation of Stupp treatment yes or no). Overall survival (OS) was defined as time from date of diagnosis (date of diagnostic surgery) to date of death or date of last follow up. Based on previous literature on the influence of antipsychotics on kynurenine metabolism^19,20^, we additionally collected information on the use of antipsychotics use at diagnosis (yes/no).

The study was approved by the Medical Ethical Committee (METC 2022-3327, MUMC Maastricht). Patient consent was waived for our retrospective study by the accredited Medical Review Ethics Committee of Maastricht UMC+, due to the fact that patients were deceased at the moment of this study and that all data collected are not traceable to individual patients (METC record 2022-3327).

Patient material was used according to the Code for Proper Secondary Use of Human Tissue (Federation of Medical Scientific Societies, The Netherlands; 2013). All experiments were performed in accordance with relevant guidelines and regulations.

### Immunohistochemistry

Tissue-microarrays (TMA) of formalin fixed and paraffin embedded (FFPE) glioblastoma tissues (n = 135) were used for immunohistochemistry (IHC). Initially, IHC was performed on whole slide sections of GBM to evaluate the expression levels of the markers. Following this, hematoxylin and eosin-stained sections were assessed by an experienced neuropathologist (JB) to identify high-density tumour areas. Three cores of 0.6 mm diameter per patient were selected and arranged in 3 µm-thick TMA sections.

Four primary antibodies targeting key proteins of the kynurenine pathway were selected: IDO1 (polyclonal, 0.1 mg/mL, cat.# NBP1-87702, Novus Biologicals, Littleton, CO), IDO2 (polyclonal, 0.45 mg/mL, cat.# OAAB08672, Aviva Systems Biology, San Diego, CA), TDO2 (monoclonal IgG_1_ OTI24A, 1 mg/mL, cat.# NBP2-45995, Novus Biologicals) and AhR (monoclonal IgG_1_ RPT1, undetermined concentration, cat.# MA1-514 Invitrogen).

The TMA sections were deparaffinized in xylene; then rehydrated in a standard series of descending alcohol immersions. Antigen retrieval was performed using EnVision FLEX Target Retrieval Solution (IDO2: pH 6.0, 10 min, 95 °C; IDO1: pH 6.0, 10 min, 110 °C; TDO2: pH 9.0, 10 min, 110 °C: AhR: pH 9.0, 10 min, 110 °C) in the decloaking chamber NxGen (Biocare Medical, inc.). To block the activity of endogenous peroxidase, the sections were incubated in EnVision FLEX Peroxidase-Blocking Reagent (5 min at room temperature). Afterwards, primary antibodies directed against IDO2 (1:350) and AhR (1:100) were applied for 20 min at room temperature. Anti-IDO1 (1:150) and anti-TDO2 (1:50) were incubated overnight at 5 °C temperature. The primary antibodies were diluted in FLEX Antibody Diluent. A signal amplification step using the Dako FLEX + rabbit or mouse Linker antibody was performed on slides which incubated with anti-IDO1 and anti-TDO2 (15 min). Then, TMA slides were incubated with EnVision FLEX/HRP for 20 min. Next, 3,3′‐diaminobenzidine (DAB, Dako; Agilent Technologies, Inc.) was utilized as the peroxidase substrate, and the sections were incubated for 10 min at room temperature. Finally, all TMA slides were counterstained with hematoxylin for 10 min at room temperature using the Leica H&E autostainer.

### Quantification of kynurenine pathway protein expression markers

**Figure 2:**
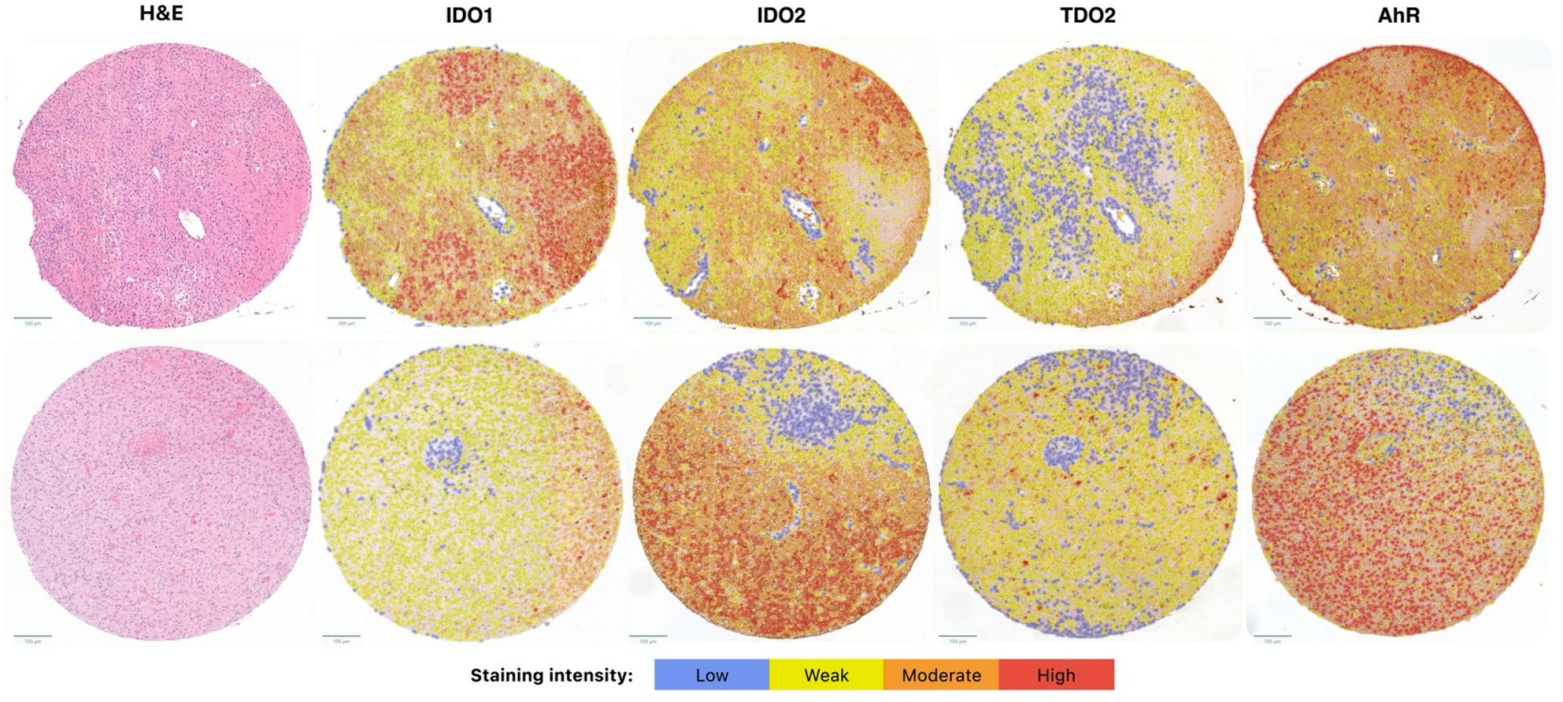
Images of two representative glioblastoma TMA cores displaying hematoxylin and eosin staining, alongside positive cell detection for IDO1, IDO2, TDO2, and AhR using IHC and immunoscoring with QuPath. Cells are color-coded according to the expression levels of key kynurenine pathway markers, based on mean DAB staining intensity—cytoplasmic for IDO1, IDO2, and TDO2, and nuclear for AhR—as follows: blue (negative), yellow (weak positive), orange (moderate positive), and red (strong positive). *H&E* Hematoxylin and Eosin, *TDO2* tryptophan 2,3-dioxygenase, *IDO1* indoleamine 2,3-dioxygenase 1, *IDO2* indoleamine 2,3-dioxygenase 2, *AhR* aryl hydrocarbon receptor.

**Figure 3:**
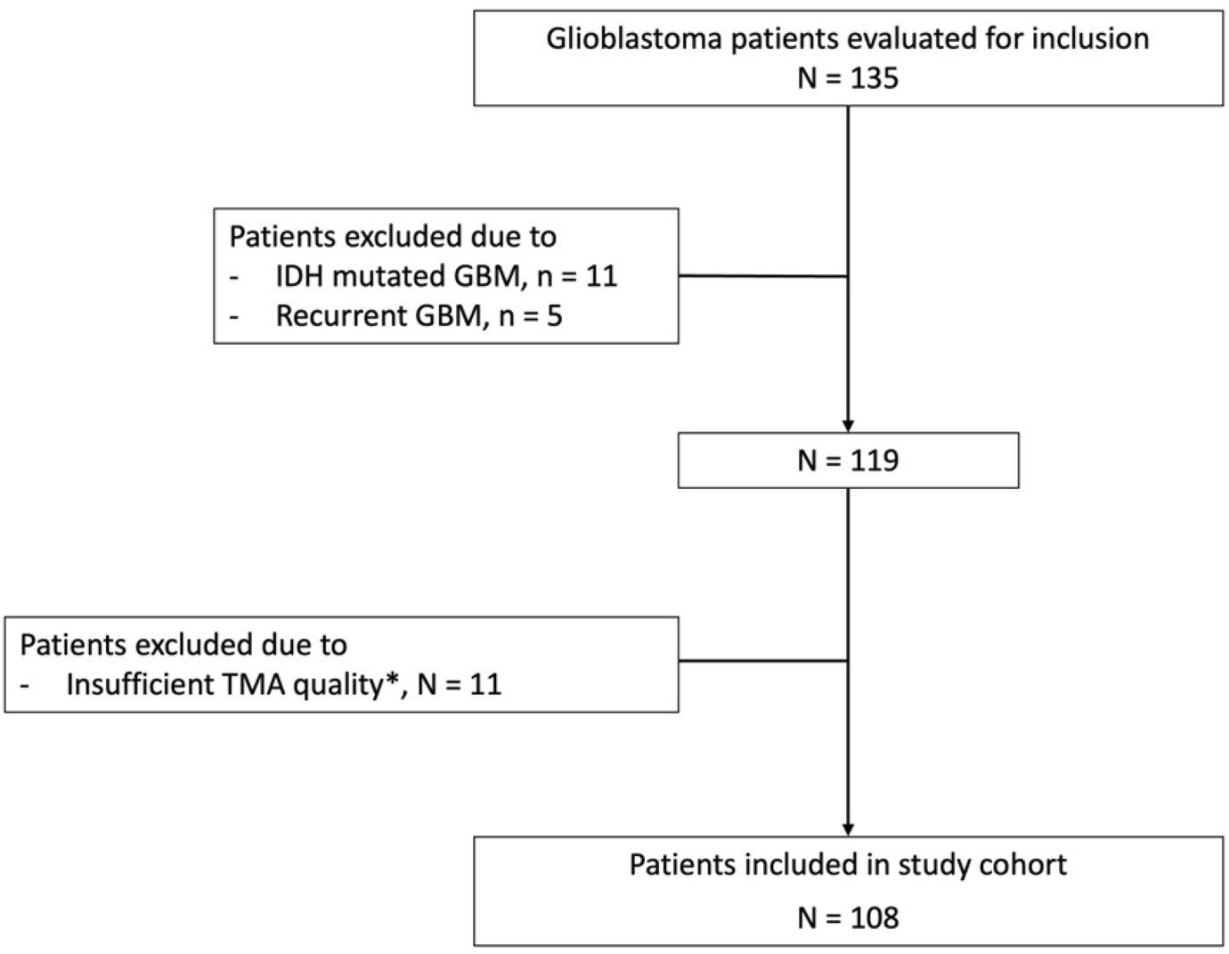
Flowchart of the patient selection process. *Reasons for exclusions were the following: insufficient tissue, N = 2; unrepresentative area for tumour tissue due to large presence of blood vessels or necrosis, N = 5; misfolded core tissue, N = 4.

QuPath version 0.2.2, a validated software for digital pathology image analysis was used for semi-automatic digital quantification of IDO1/2, TDO2 and AhR expression (Fig. 2)^21^. Tumour cells positive for cytoplasmic IDO1, IDO2, TDO2 or nuclear (AhR) were identified following a protocol adapted from previous studies^21–23^. To quantify the expression of the kynurenine markers, the histochemistry score (H-score) was calculated by QuPath^16,21,22^. The H‐score represents the extent and intensity of cytoplasmic (IDO1/2, TDO2) or nuclear (AhR) staining across each tumour core. It is calculated as follows, H‐score = 3 × % of strongly staining cells + 2 × % of moderately staining cells + 1 × % of weakly staining cells, giving a score range of 0 to 300. For each patient, a mean H-score from the three tumour cores was computed. Outliers were identified and reviewed by an experienced neuropathologist (JB) and excluded if necessary (Fig. 3).

### Data analysis

Statistical analysis was performed using SPSS version 29 (IBM Corporation, Armonk, NY, USA). A *P*-value < 0.05 was considered statistically significant. GraphPad Prism 9 was used for data visualization. Descriptive statistics were calculated for 108 patients.

X-tile was used to determine optimal cut-off values for H-scores of IDO1/2, TDO2 and AhR^24^, and divide patients into a ‘high expression’ or a ‘low expression’ group per KP marker. Kaplan–Meier analysis was performed to calculate the OS curve, and the log-rank test was applied to investigate differences in OS between patients with high and low marker expression. Next, univariate Cox-Regression analysis was used to calculate the hazard ratio (HR) for the association between H-scores below or equal to the determined cut-offs and OS. Multivariate Cox-Regression analysis was conducted using an automated stepwise forward selection procedure to assess independent prognostic variables affecting survival in our cohort. The following parameters were included: age at diagnosis, gender, MGMT methylation status, surgery type, glioblastoma treatment, corticosteroid and antipsychotics use at baseline, and ECOG Performance Score.

### Ethics approval

The study was approved by the Medical Ethical Committee (METC 2022-3327, MUMC Maastricht). Patient consent was waived for our retrospective study by the accredited Medical Review Ethics Committee of Maastricht UMC+, due to the fact that patients were deceased at the moment of this study and that all data collected are not traceable to individual patients (METC record 2022-3327). Patient material was used according to the Code for Proper Secondary Use of Human Tissue (Federation of Medical Scientific Societies, The Netherlands; 2013). All experiments were performed in accordance with relevant guidelines and regulations.

## Results

### Cohort characteristics

The final cohort for this study consisted of 108 patients (Fig. 3). Their characteristics are listed in Table 1. Mean overall survival was 14.5 months across the whole study population.

**Table 1:** Patient characteristics.

Variables	Study cohort (n = 108)
Gender, n (%)	
Male	64 (59.3)
Female	44 (40.7)
Mean age at diagnosis, years (SD)	60.3 (12.5)
ECOG score at baseline, n (%)	
0 or 1	101 (93.5)
≥ 2	4 (3.7)
Missing	3 (2.8)
Glioblastoma treatment, n (%)	105 (97.0)
Type of surgery, n (%)	
Biopsy	34 (31.5)
Resection	74 (68.5)
Corticosteroid use at baseline, n (%)	
Yes	85 (78.7)
No	22 (20.4)
Missing	1 (0.9)
Antipsychotic use at baseline, n (%)	
Yes	11 (10.2)
No	96 (88.9)
Missing	1 (0.9)
MGMT hypermethylation, n (%)	
Yes	31 (28.7)
No	76 (70.4)
Not available due to technical reasons	1 (0.9)

*ECOG* Eastern Cooperative Oncology Group Performance Status, *MGMT* 06-methylguanine-DNA-methyltransferase, *n* number of patients.

### IDO1/2-, TDO2-, and AhR-protein expression in glioblastoma tissues

Immunohistochemical analysis on glioblastoma TMA cores showed variable cytoplasmic expression of IDO1, IDO2, and TDO2, while AhR showed variable nuclear expression in tumour cells across GBM patients. The expression patterns were comparable between the three TMA cores per patient, after exclusion of non-representative areas. Staining was mostly present in high-density tumour areas, while being negative to weakly positive in regions of microvascular proliferation, vascular niches or necrosis (Fig. 2). Considering inherent tumour heterogeneity and to minimize inter-sample variation, mean H-score from the three tumour cores per patient was computed.

The H-scores were higher for IDO2 (M = 188.3, SD = 66.9) and AhR (M = 200.4, SD = 39.0) compared to TDO2 (M = 96.5, SD = 52.8) and IDO1 (M = 42.9, SD = 40.3) in GBM samples (Fig. 2). The initial immunohistochemical analysis on whole tissue sections (WTS) concurred with the expression patterns observed in the TMA cores.

### Association between tumour IDO, TDO and AhR expression and overall survival

**Figure 4:**
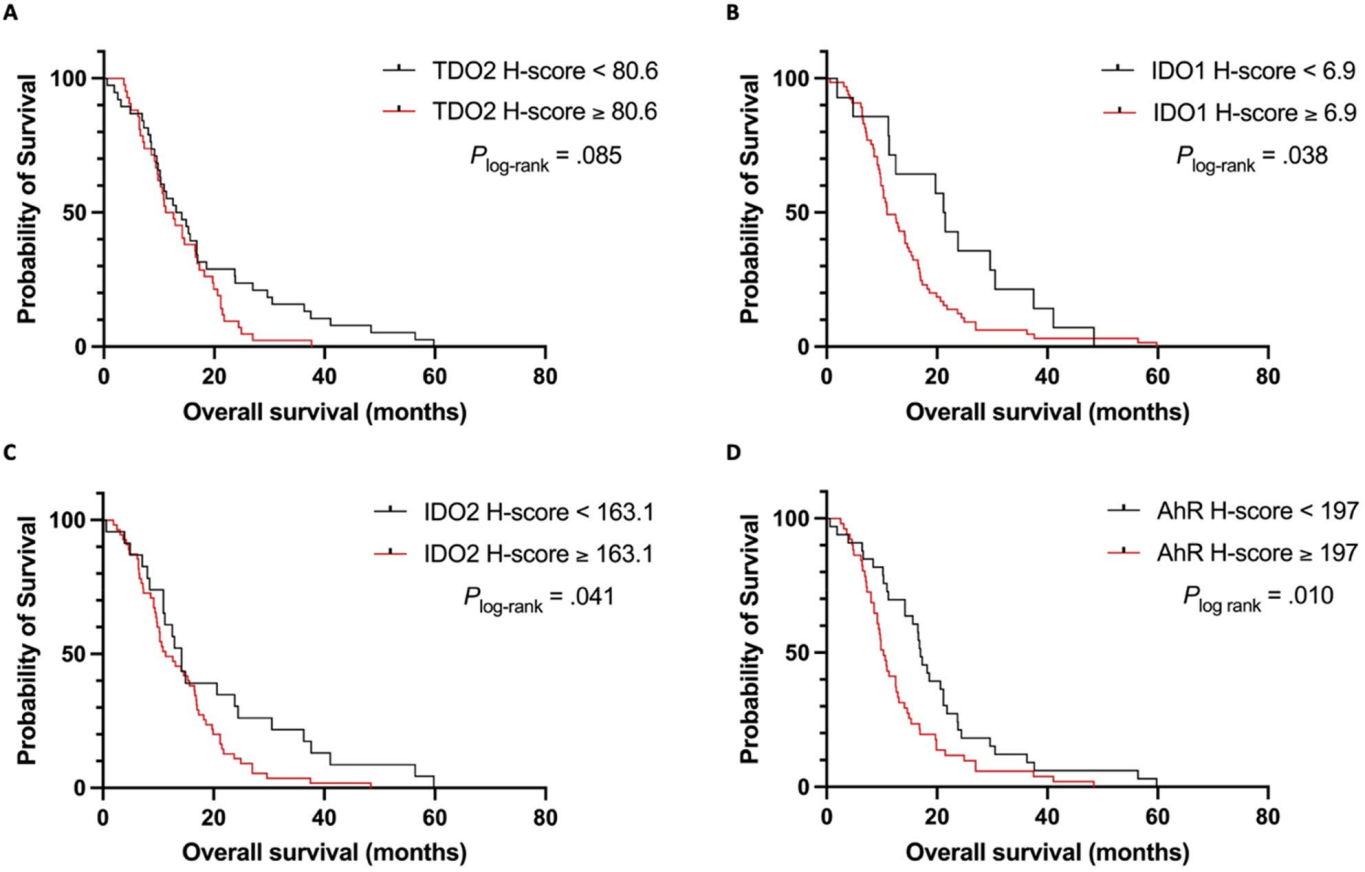
Kaplan–Meier survival curves showing the correlation between cytoplasmic TDO2 (**A**), IDO1 (**B**), IDO2 (**C**) and nuclear AhR (**D**) expression and overall survival of newly diagnosed IDHwt glioblastoma patients. Patients with H-scoring of TDO2, IDO1/2 and AhR equal or above determined cut-offs are displayed with red lines, patients below these cut-off values are shown with dark lines. *TDO2* tryptophan 2,3-dioxygenase, *IDO1* indoleamine 2,3-dioxygenase 1, *IDO2* indoleamine 2,3-dioxygenase 2, *AhR* aryl hydrocarbon receptor.

Kaplan–Meier curves for OS of patients with high- IDO1/2, TDO2 and AhR expression versus low expression are shown in Fig. 4. Patients with high cytoplasmic IDO1, IDO2 or nuclear AhR expression had a significant shorter survival compared to patients with low expression levels (log-rank test *P* < 0.05). Univariate Cox-regression analyses showed a HR of 1.852 (95% CI 1.025–3.343, *P* = 0.041) for cytoplasmic IDO1, HR 1.714 (95% CI 1.017–2.889, *P* = 0.043) for IDO2 and HR 1.800 (95% CI 1.145–2.831, *P* = 0.011) for AhR. Stepwise multivariate Cox-regression analyses confirmed that high IDO1/2 and AhR expression remained independent predictors of overall survival (IDO1: HR 3.393, 95% CI 1.707–6.748, *P* < 0.001; IDO2: HR 2.775, 95% CI 1.504–5.119, *P* = 0.001; AhR: HR 1.902, 95% CI 1.160–3.119,* P* = 0.011). Univariate and Multivariate analyses of OS in GBM patients highlighting the effects of TDO2, IDO1/2 and AhR are presented in Supplementary Tables S1–S4.

High TDO2 expression was significantly associated with decreased overall survival in multivariate Cox-regression analysis (HR 1.891, 95% CI 1.105–3.236, *P* = 0.020) but not in univariate analysis. Ad-hoc analyses revealed that GBM patients with high TDO2 expression who underwent resection had significantly better overall survival compared to those who underwent biopsy surgery (*P*_log-rank_ = 0.013; Supplementary Fig. 1).

## Discussion

The present study aimed to investigate the expression of IDO1/2, TDO2, and AhR in glioblastoma tissues of newly diagnosed patients.

Protein expression predominantly clustered within high-density tumor cell regions, notably with AhR and IDO2 demonstrating markedly elevated levels compared to IDO1 and TDO2. While prior research demonstrated a correlation between glioma grade and expression levels of these proteins, our study focused solely on GBM, revealing substantial inter-patient variability.

Here, we establish a correlation between the expression of these kynurenine pathway markers and patient prognosis.

The kynurenine pathway has gained recognition as a significant immune evasion mechanism for various malignant tumours^25–28^. To date, some studies have explored the expression of kynurenine pathway markers in gliomas^8,11,16,25,27–29^. For instance, in data derived from the Rembrandt database, patients with gliomas (WHO grades II–IV) exhibiting high expression of TDO2 and AhR had considerably lower survival rates compared to those with intermediate or low expression^11^. Similarly, analysis of data from The Cancer Genome Atlas (TCGA) found that high mRNA levels of IDO1 were associated with decreased survival in glioma^29^. Our study stands out in the field by exclusively focusing on GBM, offering a unique perspective on the expression of the kynurenine pathway markers and their prognostic implications. Unlike previous investigations, we conducted a comprehensive analysis of protein expression levels, simultaneously examining IDO1, IDO2, TDO2, and AhR. Furthermore, our utilization of digital-based pathology scoring represents a significant methodological advancement, allowing for precise interpretation of expression at the single-cell level within the histomorphological context. Our study underscores the independent prognostic significance of these markers, potentially serving as significant complements to already established prognostic factors. Of interest, elevated TDO2 expression yielded a substantial impact on survival exclusively in our multivariate analysis although it failed to reach significance in univariate analysis. This discrepancy can be attributed to the observation that GBM patients with high TDO2 expression who underwent resection had better overall survival compared to those who had biopsy surgery. This may highlights a possible compelling interaction between TDO2 expression and the surgical approach, which should be further investigated.

The dysregulation of the kynurenine pathway is thought to be responsible for a poor prognosis due to its effect on the tumour immune environment^5,6^. The upregulation of KP is linked to immune evasion of tumour cells by controlling the differentiation and effector functions of T-cells, lymphocytes, and macrophages^10–14,30^. However, the exact causal relationship between the expression of these proteins of the KP in glioblastoma and its relation to prognosis has yet not been elucidated. The analysis of comcomitant IDO1/2, TDO2, AhR expression in glioblastoma and its relationship with the tumour environment potentially will help to elucidate this interplay. A second open question is whether the dysregulation of KP is a primary phenomenon or a secondary response to other signaling pathways during gliomagenesis. The latter could be an explanation for the failure of IDO inhibitors in clinical cancer trials^31^, which might suggest that the dysregulation of the kynurenine pathway in glioblastoma may be a secondary effect rather than a primary driver. Third, there is currently limited knowledge on patient- and tumour characteristics influencing tryptophan-kynurenine metabolism in cancer. In healthy individuals, age is known to influence the KP, with increasing activity in the elderly^32^. In addition, muscle mass and exercise are linked to kynurenine levels in a healthy population, as well as certain medications such as steroids and antipsychotics^9,33–36^. To further elucidate these patient- and intrinsic tumour characteristics that influence KP specifically in glioblastoma, more research is needed.

We think that our results could already be of potential clinical importance. The introduction of these IHC markers in routine diagnostics are expected to provide useful information on prognosis already at the moment of diagnosis.

## Conclusions

Our work shows that glioblastoma patients with high expression of IDO1/2, TDO2 and AhR at diagnosis have a poorer prognosis compared to patients with low expression levels. This is independent of other known prognostic factors and probably linked to immune evasion of tumour cells. Elucidating the interplay between the concomitant expression of these proteins and the tumour microenvironment is expected to uncover potential novel therapeutic targets in glioblastoma.

### Supplementary Information

Supplementary Information.

## Data Availability

The original contributions presented in this study are included in the article and supplementary material. Further inquiries can be directed to the corresponding author. The data are not publicly available due to privacy restrictions.
