# Supplementary material for: Prognostic relevance of high expression of kynurenine pathway markers in glioblastoma
Source: Sci Rep. 2024 Jun 28;14:14975. doi: 10.1038/s41598-024-65907-3 (PMC11217262; doi:10.1038/s41598-024-65907-3)
Supplement: Supplementary file 1 — Supplementary Information. [file 41598_2024_65907_MOESM1_ESM.docx]

**Prognostic Relevance of High Expression of Kynurenine Pathway Markers in Glioblastoma – Supplementary material**

Arnaud Jacquerie^1^*, Ann Hoeben^2^, Daniëlle B.P. Eekers^3^, Alida A. Postma^4^, Maxime Vanmechelen^5,6^, Frederik de Smet^5,6^, Linda Ackermans^7^, Monique Anten^8^, Kim Severens^1^, Axel zur Hausen^1^, Martinus P.G. Broen^8^#, Jan Beckervordersandforth^1^#

#Shared co-senior authorship- these authors have equally contributed to this work.

*^1^Department of Pathology, GROW School for Oncology and Reproduction, Maastricht University Medical Centre, Maastricht, The Netherlands;*

*^2^Department of Medical Oncology, GROW School for Oncology and Reproduction, Maastricht University Medical Centre, The Netherlands;*

*^3^Department of Radiation Oncology (Maastro), GROW School for Oncology and Reproduction, Maastricht University Medical Centre, Maastricht, The Netherlands;*

*^4^Department of Radiology and Nuclear Medicine, School for Mental Health and Neuroscience, Maastricht University Medical Centre, Maastricht, The Netherlands;*

*^5^Laboratory for Precision Cancer Medicine, Translational Cell and Tissue Research Unit, Department of Imaging and Pathology, KU Leuven, Leuven, Belgium;*

*^6^LISCO—KU Leuven Institute for Single Cell Omics, KU Leuven, Leuven, Belgium;*

*^7^Department of Neurosurgery, School for Mental Health and Neuroscience, Maastricht University Medical Centre, Maastricht, The Netherlands;*

*^8^Department of Neurology, GROW School for Oncology and Reproduction, Maastricht University Medical Centre, Maastricht, The Netherlands;*

***Corresponding author:** Arnaud Jacquerie; Department of Pathology, GROW School for Oncology and Reproduction, Maastricht University Medical Centre, Maastricht, The Netherlands

E-mail address: [arnaud.jacquerie@mumc.nl](mailto:arnaud.jacquerie@mumc.nl)

**Supplementary Table S1.** Univariate and Multivariate analyses of overall survival in glioblastoma patients highlighting the effect of TDO2 expression

|  | **Univariate Cox regression** | | |  | **Multivariate cox regression (last step)** | | |
| --- | --- | --- | --- | --- | --- | --- | --- |
| **Variables** | **HR** | **95% C.I.** | **P-value** |  | **HR** | **95% C.I.** | **P-value** |
| **Gender** | .889 | .602-1.312 | .553 |  | NA | NA | NA |
| **Age at diagnosis** | 1.022 | 1.005-1.040 | **.012** |  | NA | NA | NA |
| **ECOG score at baseline** | 2.267 | 1.455-3.533 | **<.001** |  | NA | NA | NA |
| **Glioblastoma treatment** | .065 | .014-.290 | **<.001** |  | 4.970 | 2.769-8.920 | **<.001** |
| **Type of surgery** | .459 | .299-.704 | **<.001** |  | .418 | .214-.816 | **.011** |
| **Corticosteroid use at baseline** | 1.286 | .858-1.928 | .224 |  | NA | NA | NA |
| **Antipsychotic use at baseline** | .968 | .515-1.818 | .920 |  | NA | NA | NA |
| **MGMT hypermethylation** | .756 | .490-1.165 | .205 |  | NA | NA | NA |
| **TDO2 expression** | 1.505 | .942-2.403 | .087 |  | 1.891 | 1.105-3.236 | **.020** |

Variables in the univariate and final model of the stepwise forward multivariate cox-regression analysis of overall survival in glioblastoma patients highlighting the effect of TDO2 on prognosis. CI, confidence interval; HR hazard ratio; ECOG, Eastern Cooperative Oncology Group Performance Status; MGMT, 06-methylguanine-DNA-methyltransferase; TDO2, tryptophan 2,3-dioxygenase; NA, not applicable. Glioblastoma treatment encompasses treatment type and Stupp treatment discontinuation.

**Supplementary Table S2.** Univariate and Multivariate analyses of overall survival in glioblastoma patients highlighting the effect of IDO1 expression

|  | **Univariate Cox regression** | |  |  | **Multivariate cox regression (last step)** | | |
| --- | --- | --- | --- | --- | --- | --- | --- |
| **Variables** | **HR** | **95% C.I.** | **P-value** |  | **HR** | **95% C.I.** | **P-value** |
| **Gender** | .889 | .602-1.312 | .553 |  | NA | NA | NA |
| **Age at diagnosis** | 1.022 | 1.005-1.040 | **.012** |  | NA | NA | NA |
| **ECOG score at baseline** | 2.267 | 1.455-3.533 | **<.001** |  | 2.774 | 1.509-5.102 | **.001** |
| **Glioblastoma treatment** | .065 | .014-.290 | **<.001** |  | 5.091 | 2.639-9.820 | **<.001** |
| **Type of surgery** | .459 | .299-.704 | **<.001** |  | .399 | .180-.882 | **.023** |
| **Corticosteroid use at baseline** | 1.286 | .858-1.928 | .224 |  | NA | NA | NA |
| **Antipsychotic use at baseline** | .968 | .515-1.818 | .920 |  | NA | NA | NA |
| **MGMT hypermethylation** | .756 | .490-1.165 | .205 |  | .519 | .287-.939 | **.030** |
| **IDO1 expression** | 1.852 | 1.025-3.343 | **.041** |  | 3.393 | 1.707-6.748 | **<.001** |

Variables in the univariate and final model of the stepwise forward multivariate cox-regression analysis of overall survival in glioblastoma patients highlighting the effect of IDO1 on prognosis. CI, confidence interval; HR hazard ratio; ECOG, Eastern Cooperative Oncology Group Performance Status; MGMT, 06-methylguanine-DNA-methyltransferase; IDO1, indoleamine 2,3-dioxygenase 1; NA, not applicable. Glioblastoma treatment encompasses treatment type and Stupp treatment discontinuation.

**Supplementary Table S3.** Univariate and Multivariate analyses of overall survival in glioblastoma patients highlighting the effect of IDO2 expression

|  | **Univariate Cox regression** | |  |  | **Multivariate cox regression (last step)** | | |
| --- | --- | --- | --- | --- | --- | --- | --- |
| **Variables** | **HR** | **95% C.I.** | **P-value** |  | **HR** | **95% C.I.** | **P-value** |
| **Gender** | .889 | .602-1.312 | .553 |  | NA | NA | NA |
| **Age at diagnosis** | 1.022 | 1.005-1.040 | **.012** |  | NA | NA | NA |
| **ECOG score at baseline** | 2.267 | 1.455-3.533 | **<.001** |  | NA | NA | NA |
| **Glioblastoma treatment** | .065 | .014-.290 | **<.001** |  | 6.023 | 3.231-11.229 | **<.001** |
| **Type of surgery** | .459 | .299-.704 | **<.001** |  | NA | NA | NA |
| **Corticosteroid use at baseline** | 1.286 | .858-1.928 | .224 |  | NA | NA | NA |
| **Antipsychotic use at baseline** | .968 | .515-1.818 | .920 |  | NA | NA | NA |
| **MGMT hypermethylation** | .756 | .490-1.165 | .205 |  | NA | NA | NA |
| **IDO2 expression** | 1.714 | 1.017-2.889 | **.043** |  | 2.775 | 1.504-5.119 | **.001** |

Variables in the univariate and final model of the stepwise forward multivariate cox-regression analysis of overall survival in glioblastoma patients highlighting the effect of IDO2 on prognosis. CI, confidence interval; HR hazard ratio; ECOG, Eastern Cooperative Oncology Group Performance Status; MGMT, 06-methylguanine-DNA-methyltransferase; IDO2, indoleamine 2,3-dioxygenase 2; NA, not applicable. Glioblastoma treatment encompasses treatment type and Stupp treatment discontinuation.

**Supplementary Table S4.** Univariate and Multivariate analyses of overall survival in glioblastoma patients highlighting the effect of AhR expression

|  | **Univariate Cox regression** | |  |  | **Multivariate cox regression (last step)** | | |
| --- | --- | --- | --- | --- | --- | --- | --- |
| **Variables** | **HR** | **95% C.I.** | **P-value** |  | **HR** | **95% C.I.** | **P-value** |
| **Gender** | .889 | .602-1.312 | .553 |  | NA | NA | NA |
| **Age at diagnosis** | 1.022 | 1.005-1.040 | **.012** |  | NA | NA | NA |
| **ECOG score at baseline** | 2.267 | 1.455-3.533 | **<.001** |  | NA | NA | NA |
| **Glioblastoma treatment** | .065 | .014-.290 | **<.001** |  | 4.526 | 2.556-8.015 | **<.001** |
| **Type of surgery** | .459 | .299-.704 | **<.001** |  | .442 | .239-.819 | .010 |
| **Corticosteroid use at baseline** | 1.286 | .858-1.928 | .224 |  | NA | NA | NA |
| **Antipsychotic use at baseline** | .968 | .515-1.818 | .920 |  | NA | NA | NA |
| **MGMT hypermethylation** | .756 | .490-1.165 | .205 |  | .519 | .295-.911 | **.022** |
| **AhR expression** | 1.800 | 1.145-2.831 | **.011** |  | 1.902 | 1.160-3.119 | **.011** |

Variables in the univariate and final model of the stepwise forward multivariate cox-regression analysis of overall survival in glioblastoma patients highlighting the effect of AhR on prognosis. CI, confidence interval; HR hazard ratio; ECOG, Eastern Cooperative Oncology Group Performance Status; MGMT, 06-methylguanine-DNA-methyltransferase; AhR, aryl hydrocarbon receptor; NA, not applicable. Glioblastoma treatment encompasses treatment type and Stupp treatment discontinuation.

**Supplementary Figure 1**. Kaplan-Meier survival curves showing the correlation between surgery type and overall survival of newly diagnosed IDHwt glioblastoma patients, stratified by TDO2 expression.


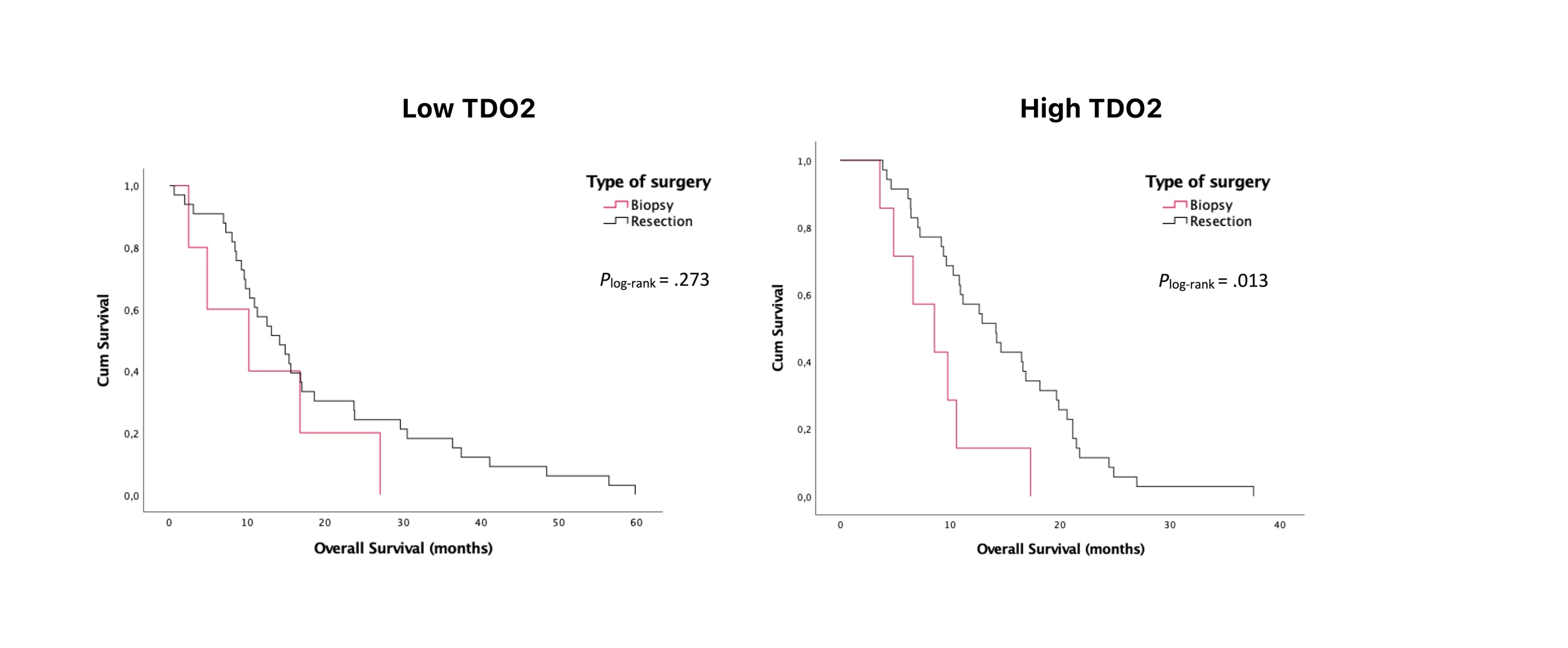


Kaplan-Meier survival curves showing the correlation between surgery type and overall survival of newly diagnosed IDHwt glioblastoma patients, stratified by TDO2 expression. Patients with H-scoring of TDO2 equal or above the determined cut-off are defined as “high TDO2” and patients below the determined cut-off are defined as “low TDO2”. Glioblastoma patients who underwent biopsy are displayed with red lines, patients who underwent resection surgery are shown with dark lines.

TDO2, tryptophan 2,3-dioxygenase
